# Architecting and Deploying IoT Smart Applications: A Performance–Oriented Approach

**DOI:** 10.3390/s20010084

**Published:** 2019-12-21

**Authors:** Ivan Zyrianoff, Alexandre Heideker, Dener Silva, João Kleinschmidt, Juha-Pekka Soininen, Tullio Salmon Cinotti, Carlos Kamienski

**Affiliations:** 1 Federal University of the ABC, Center of Mathematics, Computing and Cognition, Santo André 09210-580, Brazil; ivan.dimitry@ufabc.edu.br (I.Z.); alexandre.heideker@ufabc.edu.br (A.H.); dener.silva@ufabc.edu.br (D.S.); joao.kleinschmidt@ufabc.edu.br (J.K.); 2 VTT Technical Research Centre of Finland, FI-90571 Oulu, Finland; Juha-Pekka.Soininen@vtt.fi; 3 Advanced Research Center on Electronic Systems “Ercole De Castro” (ARCES), University of Bologna, 40123 Bologna, Italy; tullio.salmoncinotti@unibo.it

**Keywords:** internet of things (IoT), IoT architecture, IoT platform, fog computing, LoRaWAN, low power wide area network (LPWAN), FIWARE, smart agriculture, smart cities

## Abstract

Layered internet of things (IoT) architectures have been proposed over the last years as they facilitate understanding the roles of different networking, hardware, and software components of smart applications. These are inherently distributed, spanning from devices installed in the field up to a cloud datacenter and further to a user smartphone, passing by intermediary stages at different levels of fog computing infrastructure. However, IoT architectures provide almost no hints on where components should be deployed. IoT Software Platforms derived from the layered architectures are expected to adapt to scenarios with different characteristics, requirements, and constraints from stakeholders and applications. In such a complex environment, a one-size-fits-all approach does not adapt well to varying demands and may hinder the adoption of IoT Smart Applications. In this paper, we propose a 5-layer IoT Architecture and a 5-stage IoT Computing Continuum, as well as provide insights on the mapping of software components of the former into physical locations of the latter. Also, we conduct a performance analysis study with six configurations where components are deployed into different stages. Our results show that different deployment configurations of layered components into staged locations generate bottlenecks that affect system performance and scalability. Based on that, policies for static deployment and dynamic migration of layered components into staged locations can be identified.

## 1. Introduction

The internet of things (IoT) has been creating a whole new demand for system architectures, infrastructure and platform deployment approaches, in order to fulfill the requirements of a new breed of highly distributed smart applications. The number of devices connected to the Internet has been increasing steadily [1,2], so smart applications will typically have to deal with thousands or even millions of devices. Customized configurations, automated reconfigurability, and scalability are critical challenges in any successful deployment of IoT smart applications [3], in areas such as agriculture [4,5], cities [6,7], healthcare [8] and industry [9].

Layered architectures are critical structures in computer networks and distributed systems as they facilitate the understanding of roles, locations, and levels of abstraction of different networking, hardware, and software components. Different IoT architectures have been proposed for facilitating the understanding and design of smart applications, with three [10], four [11,12], or five layers [12,13,14]. The 3-layer architecture—application, network, and perception layers—was soon replaced by a 5-layer architecture by adding two new layers—middleware and business—considered thus more adequate for the rapid evolution of IoT systems. Also, intermediate 4-layer architectures have been proposed, adding, for example, a security and management layer. The reason behind the growing interest in IoT architecture is that a complete solution for IoT smart applications requires different software, hardware, and communication technologies working together and integrating a variety of platforms, components, services, and applications. IoT smart applications require careful consideration and new concepts to be developed as they are inherently distributed, spanning from sensors and actuators in the field up to a cloud datacenter and further to a user smartphone, passing by intermediary stages at different levels of fog computing infrastructure.

IoT architectures provide a functional view of software, hardware, and communication components placed into layers for facilitating system design and development. However, they do not provide indications on where these components should be deployed. As the end-to-end data flow occurring in a smart application includes sensors deployed in the field whose data usually are processed in the cloud, the deployment of architectural components is a complex task that may vary according to the characteristics, requirements, and constraints of applications and stakeholders. In such a complex environment, a one-size-fits-all approach does not adapt well to varying demands and may hinder the adoption of IoT smart applications. On the other hand, a clean and layered architecture definition might open the way towards engineering processes that enable flexible reallocation of data and services to the different deployment stages, for increased performance and scalability [15].

In this paper, we introduce a new IoT architecture for smart applications called IoTecture, comprising five layers, namely L1-Device, L2-Transport, L3-Data, L4-Model, and L5-Service. Our experience with deploying an IoT platform derived from the implementation of this architecture in four pilots with different characteristics in three countries [4] taught us that there is no one-size-fits-all approach for this process. The need for our IoTecture is due to the acknowledgment that the traditional 5-layer architecture is not prone to having an active role in the deployment of new applications since the business, application, and middleware layers are too generic with broader scopes.

Therefore, together with IoTecture, we developed the concept of an IoT computing continuum, called IoTinuum, to provide a clear view of the different deployment locations for architectural components, divided up into five stages, namely S1-Thing, S2-Mist, S3-Fog, S4-Cloud, and S5-Terminal. By representing architecture layers and deployment stages separately and mapping one into the other, we obtain a flexible mechanism for reasoning on functionality and placement, thus allowing the deployment of IoT smart applications on different scenarios. We advocate that IoTecture is not just yet another passive IoT architecture since it is actively used for the deployment of software components into distributed locations of IoTinuum.

Different deployment configurations of layered IoTecture components mapped into staged IoTinuum places generate different hardware and software bottlenecks represented by the use of system resources. In order to facilitate the understanding of the tradeoffs involved in this mapping, we conducted a performance analysis study with six configurations of a FIWARE-based IoT platform, varying the smart application (agriculture and city), the low power wide area network (LPWAN) technology [16] such as LoRaWAN [17], the workload and the use of fog computing infrastructure (no fog, lightweight fog or heavyweight fog). Our results show that different deployment configurations of layered components into staged locations generate different hardware and software bottlenecks represented by the use of system resources. The physical location where a software component is executed has a significant impact on the performance and scalability and brings different tradeoffs, as revealed by our performance analysis results. The concepts and processes involved in architecting and deploying smart applications, together with the practical performance analysis results, increase the understanding and awareness involved in the development and operation of a new breed of IoT-enabled systems.

The contributions of this paper are threefold. Firstly, the proposal of IoTecture and IoT continuum and the mapping of one into the other for facilitating the deployment of IoT smart applications. Secondly, the qualitative and quantitative evaluation of different technologies according to the IoTinuum approach, analyzing tradeoffs, and highlighting the suitability of each scenario. Thirdly, we increased the understanding of how different components of FIWARE and LoRaWAN behave under heavy workload, as our experiments indicate that some FIWARE components deal poorly with high throughput applications. This problem has not been identified before in the literature because most studies with available results do not stress the system enough to detect system bottlenecks.

In the remainder of this paper, Section 2 introduces the background and related work, and Section 3 presents IoTecture and IoTinuum. Section 4 details the research design and methods of our performance analysis study, whose results are presented in Section 5 and discussed in Section 6. Finally, Section 7 draws some conclusions and presents future work.

## 2. Background and Related Work

### 2.1. IoT Architectures and Platforms

Layered architectures are critical structures in computer networks and distributed systems as they facilitate the understanding of roles, locations, and levels of abstraction of different networking, hardware, and software components. In order to separate concerns, it is pertinent to emphasize that from an IoT architecture, different application software architectures can be derived as they define the high-level structure of a system comprising software components and relations [18]. However, here we do not elaborate on application software architectures.

The initial proposal of a generic IoT architecture had three layers, namely application, network, and perception [10]. The application layer processes large amounts of data using computational intelligence techniques and interfaces with users. The network (also called transport) layer deals with communication and networking technologies to transfer data from sensors to the place of processing and send commands back to actuators. The perception (also called sensing) layer collects the data from sensors. However, soon, a 5-layer architecture was proposed to deal with the rapid development of IoT solutions at the time, adding two more layers: middleware and business [12,13,14]. The middleware (also called processing or service) layer deals with data and databases, as well as service functions, such as discovery, composition, and management. The business layer is a loosely defined one that aggregates general functions to deal with an entire IoT system, including business models and security/privacy. Different extensions of the 3-layer IoT architecture added only one new layer, a middleware/service layer [6,19], and a security and management layer [20]. As for the latter, here we consider that orthogonal layers more adequately address these non-functional requirements.

Nowadays, most effort in developing and deploying new IoT smart applications is aimed at improving distributed data management in order to make data promptly available for the processing of application-specific models, both physical and data-driven ones. Also, these proposals follow a common approach in the past where layered IoT architectures provided an initial functional view of the entire solution, and after some iterations in the development process, they gave way to software architectures and artifacts. 

It is imperative the need to implement and deploy highly distributed IoT applications that span from the things, going through different processing/storage/transmission stages, up to the cloud, and further to the user terminal. In other words, we advocate that IoT architectures must have an active role in the deployment of new applications. An option is to embed deployment locations within the architecture, explicitly recognizing the existence of edge processing [21]. However, we consider that a new concept is needed to separate the concerns of layered software components from staged deployment locations. In the 5-layer IoT architecture, the business, application, and middleware layers are too generic with broader scopes, which do not facilitate the activity of system deployment.

An IoT platform—also known as IoT software platform [22] or IoT middleware [19,23]— implements an IoT architecture providing a variety of building blocks to facilitate the development of an IoT smart application, such as device connectivity, device management, data management, data analytics, security, and visualization. An IoT platform gathers data from IoT devices and enables the development and of smart applications that control, monitor, and manage these devices. It is often composed of several middleware components, each of then focusing on a specific feature in a particular layer to provide an end-to-end platform involving data generation, transmission, storage, and processing.

Practitioners can rely on different IoT platforms available, both open-source and commercial solutions [11,19,24]. These platforms may use multiple protocols and standards or even proprietary solutions. This plethora of platforms leads to interoperability problems since developers must adapt their applications to each platform, using specific API, information models, and protocols [25]. Thus, standard interfaces are needed to fulfill this interoperability issue among IoT applications and heterogeneous IoT platforms. Also, security requirements must be addressed, such as authentication, authorization and access control, and secure communication [26,27].

FIWARE [28] is an open-source IoT platform that has been attracting widespread attention in the last years, comprised of a series of generic enablers (GEs) that provide different services. A combination of GEs builds different applications that exchange data through a REST API based on OMA NGSI [29]. Entities and their attributes represent the key aspect of the FIWARE NGSI context management information model. 

Orion is the main FIWARE GE, a publish/subscribe context broker and an efficient data distributor, providing an interface where producers publish entities that are further notified to consumers. Orion only stores the last entity version published so that it must work together with other GEs to keep historical data. The IoT Agent GE translates sensor data in different formats to NGSI and publishes it in Orion. There are different implementations of the IoT Agent, where each one maps different IoT communication protocols and data models to NGSI. The standard FIWARE IoT Agent receives MQTT data from the sensors formatted in the Ultralight 2.0 protocol [30]—a lightweight text-based protocol aimed to constrained devices and communications where the bandwidth and device memory may be limited resources. Currently, the official FIWARE IoT Agent implementation that supports LoRaWAN communication is unstable. Therefore, we developed our own version of IoT Agent capable of mapping LoRaWAN messages in the NGSI format. 

### 2.2. LPWAN Technologies for IoT

In recent years, LPWAN technologies have been developed to meet the needs of IoT. LPWAN provides long transmission ranges, low energy consumption and low bandwidth [16], which make it an attractive technology for IoT applications that send a few dozens of bytes every couple of minutes or hours, such as street lighting, pollution monitoring and irrigation for agriculture–i.e., smart cities and smart agriculture scenarios. LPWANs follow a star topology, where sensors send data directly to a data hub called gateway, which has a stable energy source and Internet connection. Three LPWAN technologies are leading the forefront of this field: Narrowband IoT (NB-IoT) [31], Sigfox [32] and LoRaWAN [33]. NB-IoT, developed by 3GPP, uses a subset of the LTE standard that limits the bandwidth to 200kH, thus using cellular base stations for communication with devices. Sigfox uses unlicensed ISM bands to connect end-devices to base stations based on patented technologies.

LoRa operates in the sub-GHz ranges, such as 433 MHz and 868 MHz (Europe) and 915 MHz (USA and Brazil) [17]. The physical layer is called Long Range (LoRa), and the upper layers are called LoRaWAN. The latter is standardized by the LoRa Alliance [33] and can reach distances of some kilometers with a bandwidth up to 50 Kbps and a typical payload of fewer than 100 bytes. The LoRaWAN architecture comprises LoRa end-nodes (i.e., sensors and actuators) and LoRa gateways that have an active IP connection and forwards LoRa packets to a centralized server. The server is further divided into network server (deals with network issues) and application (App) server, dealing with different applications. LoRaWAN defines an ALOHA-based media access control protocol on top of LoRa communication, as well as providing security features such as authentication and cryptography. 

There are different ways of implementing the LoRaWAN architecture: (a) Open source software that can be installed in a cloud datacenter or fog according to the scenario, such as ChirpStack [34], previously called LoRa Server; (b) The Things Network (TTN), a crowdfunded online community that implements a LoRaWAN server and makes them available as a service (some services are not free, though) [35]; (c) Proprietary solutions, such as Loriot [36]; (d) The development of a new implementation that means in practice reinventing the wheel.

### 2.3. Fog and Mist Computing for IoT

Fog computing is a new approach for dealing with the enormous amount of data generated by IoT smart applications [37]. It addresses three key challenges: (a) decreasing latency for real-time application; (b) reducing data traffic between devices and cloud, and; (c) providing a load balancing alternative to soften the processing burden of the cloud. The fog metaphor is similar to the cloud one, but the former is closer to the ground and the people than the latter [38]. The underlying fog infrastructure is a virtualized platform that provides computing, storage, and communication services between the users and the cloud datacenter [39]. In other words, fog is the distributed computing infrastructure for IoT. Here we consider fog computing and edge computing as interchangeable terms, even though minor differences might exist [40].

While the fog aims at reducing bandwidth usage, latency, and data communication with the datacenter, mist computing extends this concept further in IoT-based systems, closer to the sensors and actuators [12]. Although there is no consensus about the definition of mist, some common understanding can be found, such as being located closer to the devices [21,41]. Mist can be considered the lowest level of a hierarchical fog computing system.

Usually, mist nodes host communication facilities, such as a LoRaWAN gateway or a Wi-Fi access point. Additionally, they can also host light processing and temporary storage, typically using ARM processors, such as Raspberry Pi and similar. According to the Internet Engineering Task Force (IETF), these devices belong to a category of general-purpose constrained nodes, making the difference between sensing devices based on microcontrollers [42]. The mist infrastructure may be composed of different nodes working together to provide services to devices within the same geographical area, which not necessarily are packed into the same box.

### 2.4. IoT Smart Applications

The IoT allows people and objects to be connected at any time, from anywhere, using standard networks and protocols to access innovative smart services. Sensors of different natures in different spaces coupled with ubiquitous GPS-equipped smartphones, in addition to the resources available in core or edge datacenters, allow seamless automation of typical manual activities. The use of a variety of combined technologies—such as IoT, cloud computing, big data analytics (e.g., machine learning techniques) and network softwarization-make it possible to build a myriad of new smart applications to the benefit of our society.

Here we use four IoT verticals as examples of smart applications, namely smart cities, smart agriculture, smart healthcare, and smart industry. Although other smart applications exist, these four ones represent the majority of currents efforts where IoT has been applied in real cases [3]. Cities are becoming smarter for citizens and municipal governments, as new and existing technologies are increasingly used to the development of a substantial variety of services and applications [6,7]. In farms, the use of technology to add intelligence to agriculture still has a vast potential to generate significant advances. Smart agriculture (or more broadly smart farming), depends on innovative technologies to increase the productivity of crops and animal products, to make efficient use of precious resources like water, and to decrease the amount of chemical substances used (e.g., medicines and pesticides) [5]. Notably, we have been involved in using IoT and machine learning solutions within the SWAMP project, which develops IoT-based approaches for smart water management for a precision irrigation application, and pilots them in Italy, Spain, and Brazil [4]. Healthcare is an essential area for introducing IoT, given its enormous potential to provide quality of life to the society in applications such as elderly monitoring, chronic diseases, fitness programs, and remote treatment [8]. Finally, industrial automation has been experiencing a significant change in the last years, which is due to the recent technological advances that allow more profound interconnection and improved integration and production control [9].

A critical concern in the development of IoT smart applications is security [26,27,43]. The applications must deal with data privacy, confidentiality, integrity, authentication, and availability of services. The four IoT verticals have similar security requirements to protect the data. In healthcare, privacy means the health records of the patients must be appropriately secured, using adequate authentication methods to avoid access by non-authorized users, and secure communication for exchanged data [27]. The data integrity is also important since incorrect sensor measurements or prescriptions can cause serious health problems to the patient. Most embedded sensors and wearables used in healthcare have resource limitations, which makes it challenging to use traditional security mechanisms, usually computationally expensive. Since many IoT devices are limited in power, processing, and memory, this is a challenge also for applications in smart cities, smart agriculture, and smart industry.

### 2.5. Performance and Scalability of IoT Systems

Different forecasts have been reporting the availability of billions of connected devices in the next years [1,2]. Since smart applications will typically have to deal with thousands of devices-or considerably more-performance and scalability concerns are key to any successful IoT deployment. Yet, most current reported experiences show: (a) small-scale pilots [7,44]; (b) simulation-based or analytical results [21,45]; (c) measurement-based results with limited scope [19,46]; (d) no quantitative results at all [2,3,5,8,9,11,12,16,24,37,47]. Also, different architectural and deployment choices for IoT systems affect scalability, and real-time decision-making is possible in an environment composed of thousands of sensors [4,48]. Therefore, understanding the tradeoffs involved in planning and deploying different software components of a specific scenario on different location infrastructures-such as cloud, fog, or mist—requires careful consideration of the performance and scalability of the solution.

Cruz et al. [19] proposed qualitative and quantitative metrics and evaluated the performance of various IoT platforms. Out of 11 platforms initially analyzed by the qualitative approach, five were selected for the performance analysis. However, since they adopted a generic approach, the authors did not go into the specifics of individual platforms, and they did not evaluate different deployment infrastructures.

Martínez et al. [46] gave a detailed description of a FIWARE testbed architecture configured for precision agriculture, which differs from our approach, mainly because our focus is to test different configurations considering our IoTecture and IoTinuum. Besides, their test application connects directly to Orion with NGSI JSON, while we used an IoT Agent with an IoT sensor simulator that generates synthetic data. 

This paper differs from previous work showing a performance analysis study of six deployment configurations of FIWARE and LoRaWAN/LPWAN for smart agriculture and smart city, involving different placement strategies of components over fog and cloud infrastructures. It extends previous preliminary performance results presented in [4] and [48], which focused on more specific scenarios of smart irrigation.

## 3. Architecting and Deploying IoT Smart Applications

**Figure 1:**
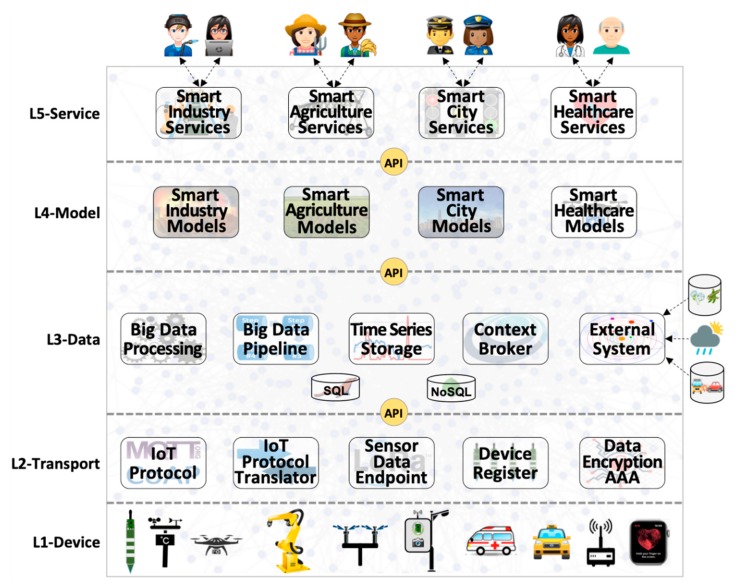
IoT architecture (IoTecture) for smart applications.

A complete solution for IoT smart applications requires different software, hardware, and communication technologies working together and integrating a variety of platforms, components, services, and applications. These technologies play different roles and provide functions that operate in different perspectives of the scenarios addressed by smart applications. Figure 1 depicts our proposed IoTecture, a generic 5-layer architecture based on our previous experience with the development of IoT smart applications [4,49]. We recognize that a myriad of different layered architectures may be conceived having in mind different target applications.

Nevertheless, IoTecture is the outcome of both an analysis of the literature (Section 2) and an iterative refinement process, and as such, it is effectively used in the specification of pilot deployment scenarios, as well as performance analysis studies. Compared to the 5-layer IoT architecture [12,13,14], our 5-layer one explicitly adds support for highly distributed data management functions and separates physical and data-driven models from application services. Also, by clearly identifying and separating components that are logically bound to the data, model, and service layers, IoTecture actively helps the deployment of IoT smart applications over different distributed locations in the mist, fog, and cloud. From a software architecture point of view, components of different layers are implemented by services—actually encapsulated into Docker containers with an exposed API, therefore microservices. Components inside each layer serve as representative examples of different alternatives that may vary according to characteristics, requirements, and constraints of applications and users.

- Layer 1 (L1-Device): Sensors, actuators, and communication technologies—both wired and wireless—make up the lowest layer of the IoT architecture. Devices are sensors and actuators that represent IoT things. Figure 1 depicts some examples of Layer 1 components, such as industrial robots, that have many sensors to track their actions, and actuators to control engines that make them move according to different degrees of freedom, both exchanging messages with a smart industry application via wired and wireless communication technologies.
- Layer 2 (L2-Transport): Collecting data from sensors, making it available to data management functions, receiving commands from application models, and sending them to actuators require a good deal of intermediate components to make it happen. Figure 1 depicts some examples of generic data transport functions for sensing and actuation purposes, such as: (a) IoT protocol (e.g., MQTT [50]) that sends data from IP speaking devices to place where they are transformed or filtered either by other components of this layer or by data management components; (b) IoT protocol translator (e.g., FIWARE IoT Agent) converts data from IoT protocol format (e.g., byte stream for MQTT) into its internal format within data management components (e.g., NGSI/JSON [29] for FIWARE) and vice-versa; (c) Sensor data endpoint (e.g., ChirpStack [34]) transports, unpacks and decodes data for specific IoT wireless communication technologies such as LPWAN [16]; (d) Device register deals with the myriad of connected devices; (e) Data encryption and Authentication, Authorization, and Accounting (AAA) is pervasively necessary for IoT communications and included in Layer 2. A general function of data security and network/service management are transversal functions, as all layers pervasively needed them (not represented in Figure 1, though).
- Layer 3 (L3-Data): Every stage of any end-to-end IoT data flow needs to deal with data in different ways, such as storing, retrieving, distributing, transforming, and filtering. Figure 1 depicts some examples of generic data management functions, such as: (a) Context broker (e.g., FIWARE Orion [51]) for context data distribution and related storage system; (b) Big data pipeline (e.g., Apache Kafka [52]) for raw data distribution and replication; (c) Big data processing (e.g., Apache Spark [53]) for processing large amounts of data; (d) Time series storage (e.g., CrateDB [54]) for historical data, and; (e) Interfacing with external systems, such as weather forecast services, online city traffic management services and databases.
- Layer 4 (L4-Model): Represent application-specific models for any type of processing over data collected from sensors and external systems, encompassing an assortment of algorithms, equations, methods and techniques that change the data into knowledge that is used by end-users to change the environment in a way that fits their best interests. For example, a smart irrigation application may use soil and weather data, as well as weather forecast information (external system), to feed physical and machine learning models to precisely compute when and how much crops should be irrigated [4].
- Layer 5 (L5-Service): Contains services that support the interfaces and the interaction with end-users of smart applications. This layer encompasses all services, applications, and graphical interfaces that provide visualization of sensor data, sensing and actuation infrastructures, analyses, choices, and decisions, as well as commands to change the state of the system. Figure 1 depicts four IoT verticals as examples of smart applications, namely smart agriculture, smart city, smart healthcare, and smart industry.

IoTecture, depicted by Figure 1 provides a high-level structural view of software, hardware, and communication components placed into layers for facilitating system design and development. However, it does not provide indications on where these components should be deployed–i.e., mist, fog, or cloud-in a naturally distributed IoT smart application. As the end-to-end data flow occurring in a smart application includes sensors deployed in the field whose data usually are processed in the cloud, the deployment of architectural components is a complex task that may vary according to the characteristics, requirements, and constraints of applications and stakeholders.

**Figure 2:**
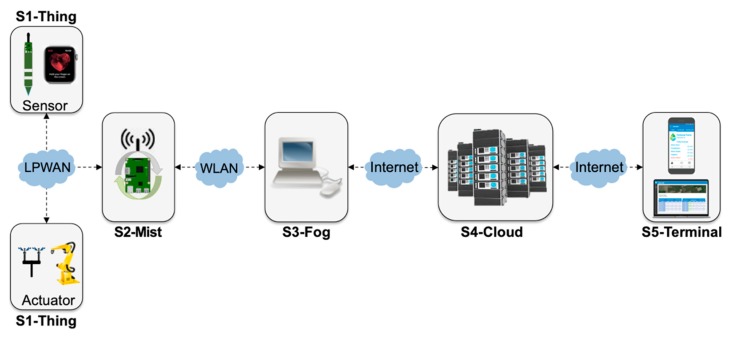
The IoT computing continuum (IoTinuum).

In order to provide a clear view of the different deployment locations for architectural components, we developed the concept of an IoT computing continuum (shortened to IoTinuum), shown in Figure 2. IoTinuum is divided up into stages or deployment locations, which may vary according to characteristics of the existing infrastructure, and extends the concept of IoT-fog-cloud Continuum [47]. IoTinuum is an approach of formalizing the highly distributed infrastructure of IoT systems and facilitating the creation of different deployment views for the mapping between layered architectural components into staged locations. For the sake of this paper, we identify five stages: S1-Thing, S2-Mist, S3-Fog, S4-Cloud, and S5-Terminal.

- S1-Thing: represents the stage implemented by hardware devices, i.e., sensors and actuators, which convert analog to digital signals and perform simple device-specific transformations, such as calibration.
- S2-Mist: mist nodes are installed in the field and play the role of radio gateways [55] in the context of LPWAN—or similar technologies—that support device data communication but also processing, such as data aggregation. Mist nodes are close to the devices they assist and typically have modest computing resources, similar to a Raspberry Pi.
- S3-Fog: fog nodes are installed in sheltered places with stable power supply and include equipment such as laptops, desktops, or small servers, which provide system reliability, robustness, resilience, and low latency for time-sensitive applications.
- S4-Cloud: public or private clouds host physical servers and virtual machines in a datacenter with plenty of resources. There is a noticeable increase in the processing power from S1-Thing to S4-Cloud
- S5-Terminal: the place where the end-user interacts with a smart application, connected to S4-Cloud, but also S3-Fog in some configurations.

The five stages of IoTinuum define the end-to-end information path, starting with data collected by sensors up to commands executed by actuators. These five stages might not be necessarily present in all configuration scenarios. Instead, depending on application characteristics, requirements and constraints, S2-Mist, S3-Fog, or S4-Cloud stages may not be present. Communication technologies between S1-Thing and S2-Mist are usually LPWAN (e.g., LoRaWAN), and between S2-Mist and S3-Fog are usually WLAN (e.g., Wi-Fi).

**Figure 3:**
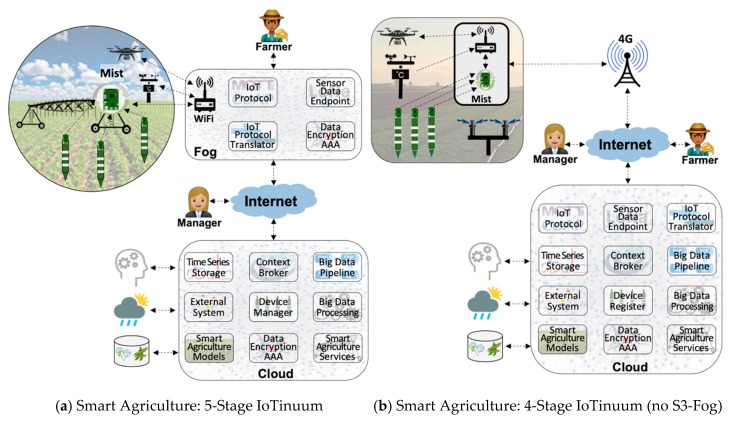

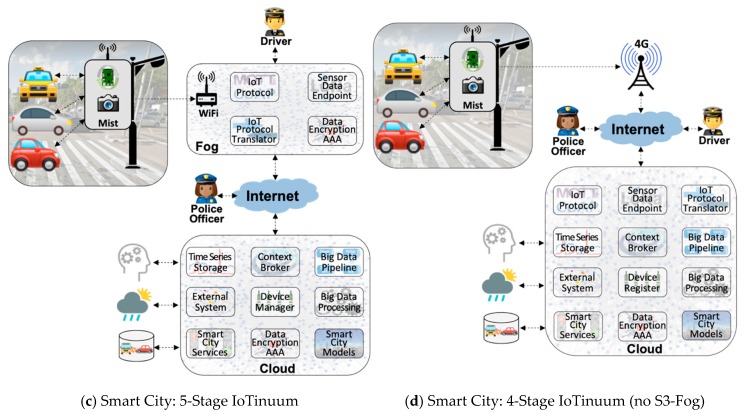
Smart applications for deploying IoT architecture components on different configurations of IoTinuum.

Figure 3 represents different mappings of the IoT architecture into two deployment views or configurations of IoTinuum-with and without S3-Fog—for the smart agriculture and smart city scenarios. The end-users implicitly represent S5-Terminal. Figure 3a depicts a scenario of a smart irrigation based on a center pivot that sprays water on a circular plot where the S2-Mist is placed. L2-Transport components of the architecture are located in S3-Fog-placed in the farm office-and L3-Data, L4-Model, and L5-Service layers are located in S4-Cloud. Figure 3b depicts a sprinkler irrigation scenario where S2-Mist is located in an environmentally protected box in the field. S3-Fog is not used by the choice of the farmer, and therefore all remaining components are located in S4-Cloud. Figure 3c,d depict similar configurations for smart traffic control. For both cases, S2-Mist is located in a lamppost, whereas for Figure 3c, S3-Fog is located in an aggregation point (a point of presence of the electricity company).

**Figure 4:**
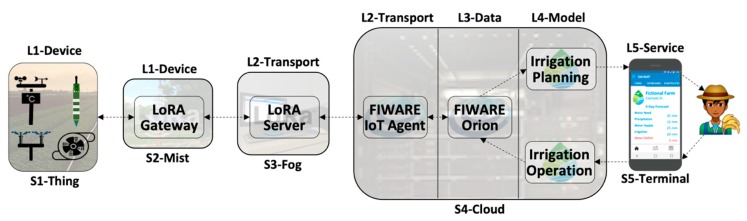
Mapping of the 5-layered IoTecture over the 5-staged IoTinuum for a smart irrigation scenario.

Figure 4 further explains the mapping between architecture and continuum with a Smart Irrigation scenario from the SWAMP project [4] with a deployment configuration that combines components of the five layers of IoTecture distributed over the five stages of IoTinuum. For this simplified example, L1-Device contains sensors (soil moisture sensor and weather station) and actuators (pump and sprinkler) in S1-Thing and a LoRaWAN Gateway in S2-Mist. Both stages are installed in the farm field. L2-Transport is deployed into S3-Fog with the LoRaWAN server (sensor data endpoint), as well as in S4-Cloud with FIWARE IoT Agent (IoT protocol translator). L3-Data is deployed only in S4-Cloud, represented by FIWARE Orion. L4-Model is also deployed in S4-Cloud, represented by specific models for irrigation planning and operation. The application frontend of L5-Service runs in S5-Terminal (SWAMP application), which is accessed by farmers via their smartphones. The application backend runs in S4-Cloud, although not represented in Figure 4.

Two important observations can be made about the scenario depicted in Figure 4. Firstly, it is only one example among different ways of deploying architectural components (IoTecture layers) over deployment locations (IoTinuum stages). For example, in a scenario where the farmer does not desire any on-premises infrastructure, S3-Fog disappears, and the LoRaWAN server could be deployed in S4-Cloud. Secondly, a real smart irrigation application demands more components that are not in Figure 4, which appear in Figure 3 represented by generic function names. By representing architecture layers and deployment stages separately and mapping one into the other, we obtain a flexible mechanism for reasoning on functionality and placement and being able to provide deployment views to IoT smart applications on different scenarios. We consider it a more robust approach compared to mixing both concerns in a single architecture, such as the one proposed by Asif-Ur-Rahman et al. for smart healthcare applications [21].

## 4. Performance Analysis: Design and Methods

As shown in Section 3, the mapping between components of the layered IoTecture into stages of the IoTinuum may result in different deployment configurations, depending on the characteristics, requirements, and constraints of applications and users. The performance of smart applications is influenced by different end-to-end sequences of connected software, hardware and communication technologies through which the data flow has to pass in its way from sensors (S1-Thing) up to the cloud (S4-Cloud)–and user (S5-Terminal)-and back to actuators (S1-Thing). Depending on where components are deployed and how they are connected, the bottlenecks may move from one place to the other, as well as the amount of data to be processed, stored, and transmitted may increase or decrease, thus impacting performance metrics, such as response time. In this section, we conduct a performance analysis study with six configurations to help us understand the performance tradeoffs of different architectural and deployment choices, based on our IoTecture and IoTinuum presented in Section 3.

### 4.1. Design Decisions and Deployment Configurations

**Figure 5:**
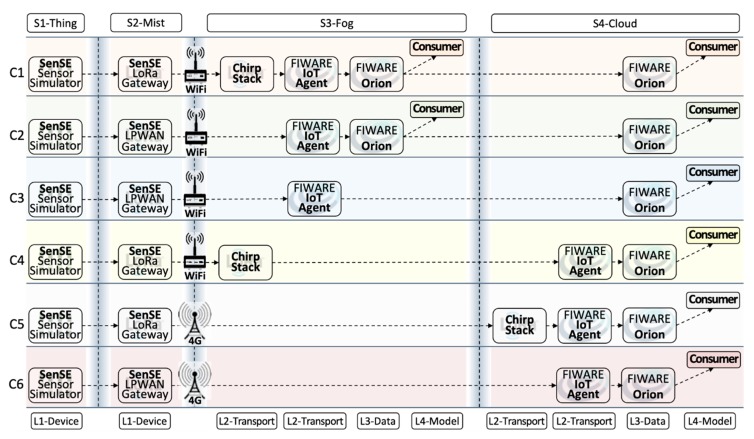
Deployment configurations of layered IoTecture components into staged IoTinuum places.

**Figure 6:**
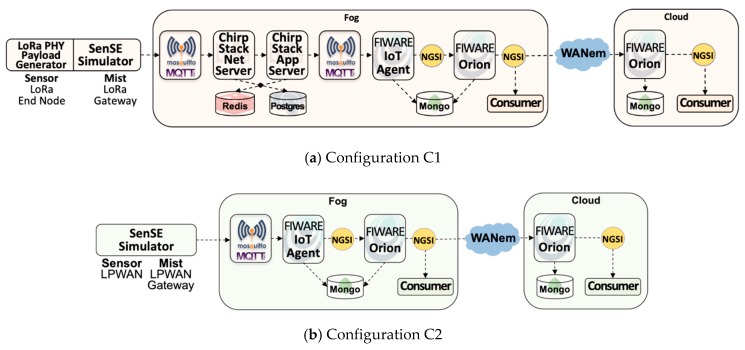

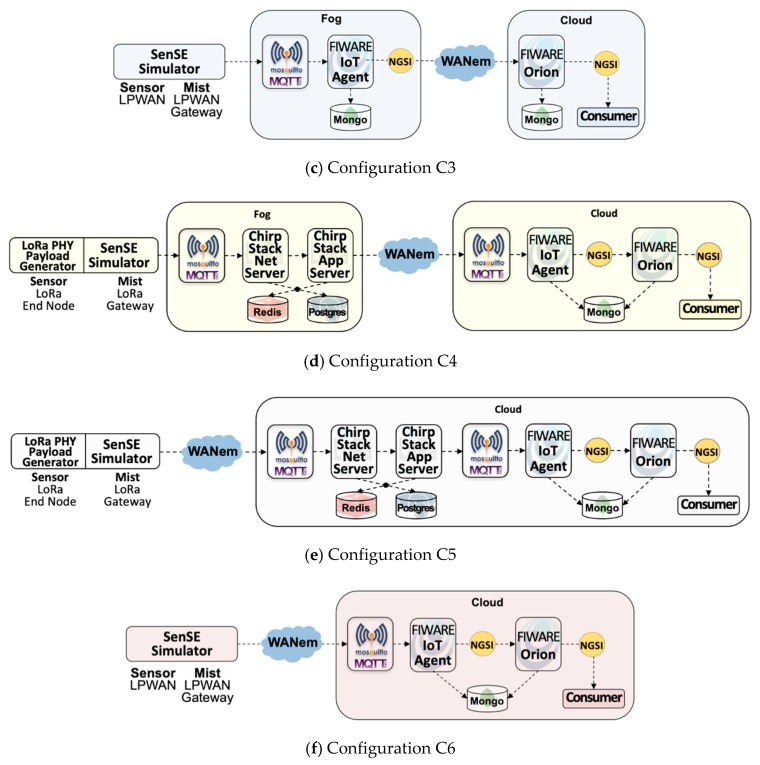
Testbed setup for the six deployment configurations.

The scope of this performance analysis study follows design choices focused on typical IoT platform components, independent of application and communication technologies. Figure 5 depicts six deployment configurations of layered IoTecture components into staged IoTinuum places devised following four key design choices:

- Application independence: Layers L4-Model and L5-Application of IoTecture represent specific applications. This study addresses architectural components that comprise a common IoT platform, oblivious of smart application particularities. Thus, application-specific components are not included in our experiments. As applications must consume data managed by L3-Data, a simple Consumer component was added to represent L4-Model as a generic data sink. As a direct consequence of this choice, data flows only in one way, from sensors to the consumer located at the cloud. In other words, users are not involved, and therefore, L5-Service and T5-Terminal are outside of the scope of this work.
- Communication independence: in order to understand scalability limits, our study was performed in a lab testbed, wherein we can increase the workload by abstracting a large number of sensors using the SenSE sensor simulator [56] that generates data at different rates. Therefore, real IoT communication technologies-e.g., LPWAN-were not included in our experiments. Nevertheless, since LoRaWAN is currently a key LPWAN technology that needs a middleware component to work properly, a LoRaWAN server was included, as it belongs to L2-Transport playing the role of a sensor data endpoint. SenSE generates data emulating a LoRaWAN device, in a way that the server is unaware of not receiving data from real sensors. The configurations with ChirpStack are compared to configurations with a generic LPWAN technology where the sensor simulator generates data in a simple string-based protocol–e.g., Ultralight 2.0-and does not need to go through to the particularities of a specific LPWAN technology.
- Scenario simplicity: to clearly understand the effect of different deployment configurations-i.e., the mapping of IoTecture components into IoTinuum stages-a minimum set of components was used to guarantee a data flow that starts at sensors-actually, at SenSE-and ends at the consumer. The set of software components included in the evaluated IoT platform is composed of (Table 2): SenSE sensor simulator, ChirpStack, FIWARE IoT Agent, FIWARE Orion, and consumer. They include other auxiliary components as MQTT broker and databases (MongoDB, Redis), represented in Figure 6.
- Fog Dilemma: S3-Fog may frequently leave behind in specific configurations for different reasons, such as the choice of the farmer in not hosting IoT infrastructure within the farm in locations with stable and high-speed 4G connections. S2-Mist is required in most cases, especially for LoRaWAN. Also, we assume that S4-Cloud is always present. Therefore, we tested different configurations, with and without S3-Fog, and with heavyweight fog-local processing components-and lightweight fog-only communication components.

Table 1 further explains the six deployment configurations that resulted from these four choices, called C1, C2, C3, C4, C5, and C6. They were obtained by varying two factors, namely LPWAN technology (LoRaWAN vs. Generic LPWAN) and fog dilemma (no fog vs. heavyweight fog vs. lightweight fog.

- Configurations C1, C2, C3, and C4 contain S3-Fog, whereas configurations C5 and C6 preclude it. It also implies that the former configurations assume Wi-Fi WLAN connectivity between S2-Mist and S3-Fog, while the latter assumes a 4G connection. The difference between Wi-Fi and 4G here is related to the link characteristics emulated by the WANem WAN emulator [57].
- Configurations C1, C4, and C5 use LoRaWAN, while configurations C2, C3, and C6 assume a generic LPWAN technology–e.g., the physical LoRa modulation.
- Configurations C1 and C2 have a heavyweight fog, which means that data are processed and consumed there with low latency. It also means that since we are using FIWARE Orion as the primary data distributor, one Orion in S3-Fog must connect to another Orion in S4-Cloud. Since Orion is a publish/subscribe context broker, the S4-Cloud Orion subscribes to the S3-Fog Orion, and whenever a message is published, the latter notifies the former. On the other hand, configurations C3 and C4 are based lightweight S3-Fog versions running only L2-Transport components, where C3 only has ChirpStack, and C4 only has the IoT Agent.

The main components used in the six deployment configurations are depicted in Table 2.

### 4.2. Experimental Design

Figure 6 extends Figure 5, providing additional technical information for the six deployment configurations introduced in, needed for the clear understanding of both experiments and results.

- Smart applications: two scenarios of smart agriculture and smart city are considered in our experiments. The critical difference between them is the approach for sensor data generation. In the smart agriculture scenario, we consider many soil moisture sensor probes, continually generating and transmitting one data packet every 10 min. In the smart city scenario, we consider vehicles playing the role of sensors that communicate with a traffic light according to a Poisson arrival rate. In both scenarios, the SenSE sensor simulator synthetically generates sensor data.
- LPWAN Technology: data is generated by SenSE using the Ultralight 2.0 protocol format in both scenarios. Also, for LoRaWAN, sensor data packets are coded according to the LoRa PHY format and encrypted according to the AES algorithm determined by the LoRaWAN standard. For Generic LPWAN, plain sensor data is transmitted directly to the receiving end, which is always an MQTT broker. There is a sizeable difference in the payload generated by SenSE in the configurations with LoRaWAN and generic LPWAN. With LoRaWAN, SenSE sends ChirpStack a JSON over MQTT message with information about the emulated LoRa modulation in addition to the actual encrypted payload, composing a message of 314 bytes. In the configurations without LoRaWAN, SenSE sends an Ultralight 2.0-structured MQTT 65-byte message to the IoT Agent, significantly smaller than the LoRa message
- S1-Thing and S2-Mist: the SenSE simulator abstracts L1-Device in both S1-Thing and S2-Mist for all configurations. In other words, since our experiments are controlled and performed in a testbed, L1-Device is not present, except for the LoRa PHY format that is generated by SenSE, in order to understand the real effect of ChirpStack.
- ChirpStack: an open source LoRa server composed of two main components, the ChirpStack Network Server and the ChirpStack application server. Both communicate via MQTT with each other and with sensors and actuators, as well as platform components, e.g., FIWARE IoT Agent. Also, they use Redis and PostgreSQL as databases.
- WAN Emulation: to emulate the network link between the fog and the cloud, we used a WAN emulator (WANem) with 45 ms of latency and 5 ms of jitter. We defined the parameters to input in the WANem through a simple experiment performed the ping command to a VM located in the Amazon cloud in São Paulo, sent through 4G.
- Infrastructure: experiments were performed in an OpenStack-powered private cloud. As experiments were in a testbed, they are not influenced by other virtual machines running in the same physical servers. VMs for cloud and fog are based on standard Amazon AWS configurations: cloud runs on a t2.medium instance (2vCPU-4GB of RAM) and fog on a t2.small instance (1vCPU–2 GB RAM). Our testbed was composed of two physical servers with the following configuration: Intel(R) Xeon(R) CPU E3-1240 V2 @ 3.40GHz-8 cores and 16 GB of RAM.

Experiments with a large number of sensors-representing soil moisture sensors or cars passing through a traffic light-simultaneously send messages to the platform, in order to verify and understand scalability tradeoffs. We varied all levels of all factors in each scenario, consisting of 36 different possibilities (Table 3).

Notice that the workload for smart agriculture and smart city scenarios is specified in different units since they are based on different probability distributions for time-driven and event-driven sensors, respectively. Both represent somewhat similar workloads: 500–1000–1500 messages per minute for the smart agriculture scenario, and 480–960–1440 average messages per minute for the smart city scenario. Each experiment took 2 min with 30 replications, totalizing 36 h.

### 4.3. Metrics

The experiments collected two categories of metrics:

- Elapsed Time: The average time taken since a sensor data point is generated by SenSE until the consumer receives it. This metric represents how long it takes for sensor data to be available to any subscribed application. We present the Thing-to-Cloud Elapsed Time that encompasses the end-to-end path taken by data from its inception by SenSE in S1-Thing up to its consumption in S4-Cloud, and Thing-to-Fog Elapsed Time specifically for configurations C1 and C2 that contains an L4-Model Consumer application in S4-Cloud.
- System metrics: CPU and RAM usage per Docker container, which allows observing each component, located in S3-Fog or in S4-Cloud, collected every 5 s.

## 5. Performance Analysis: Results

**Figure 7:**
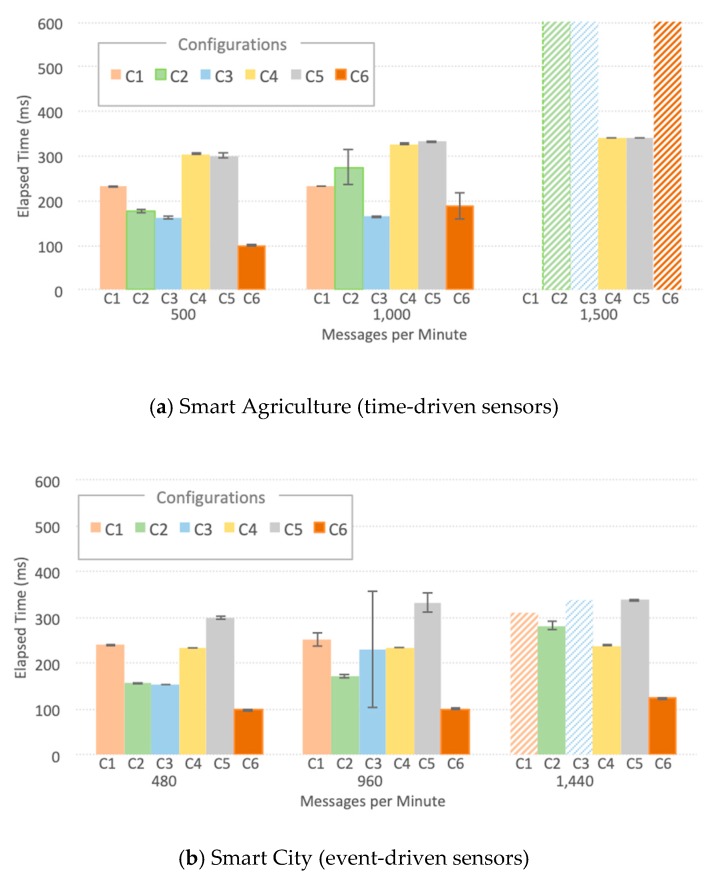
Device-to-Cloud elapsed time for IoT smart applications.

Figure 7 summarizes the key results of Thing-to-Cloud Elapsed Times for smart agriculture and smart city for the six configurations C1, C2, C3, C4, C5, and C6. The bars filled with horizontal, diagonal and zigzag patterns mean that the experiments did not finish correctly due to higher workloads, even after numerous repetitions. Also, configuration C1 did not run at all for smart agriculture with 1500 messages per minute-15,000 sensor probes-as VMs always failed shortly after the beginning of the experiments.

Comparing both scenarios, we can observe that smart city (Figure 7a) had a slightly better result, especially for the highest workload of each scenario. Those results show that the components tested performed better with messages arriving in bursts-event-driven sensors-instead of messages arriving with a constant flow-time-driven sensors. Notably, configurations, including LoRaWAN (C1, C4, and C5), achieved higher scalability than the ones with generic LPWAN. Besides performing its intrinsic function, ChirpStack works as a cushion for sensor data and softens message spikes. Regarding an overall comparison of the experiments using LoRaWAN (configuration C1, C4, and C5) and generic LPWAN (configuration C2, C3, and C6), it can be noted that for lower workloads ChirpStack adds about 200 ms to the processing, since more software components are needed for addressing issues such as security and authorization.

In the smart agriculture scenario with the highest workload (1500 messages per minute), only two configurations ran up smoothly to the end of the experiments without failing, namely C4 and C5. This result was not expected since C6 failed, and it is the lightest configuration in terms of the number of software components that are placed in the robust VM in the cloud. Nevertheless, both scenarios highlight the stability and scalability of configurations C4 and C5, mainly due to the improved data flow control of ChirpStack. On the other hand, when the workload is placed directly on the IoT Agent, the experiment fails, e.g., C6.Configurations C1 and C2 use a heavy fog, where the Consumer uses data directly in S3-Fog, allowing applications to process part of the data closer to the user and thus to respond to changes in real-time.

**Figure 8:**
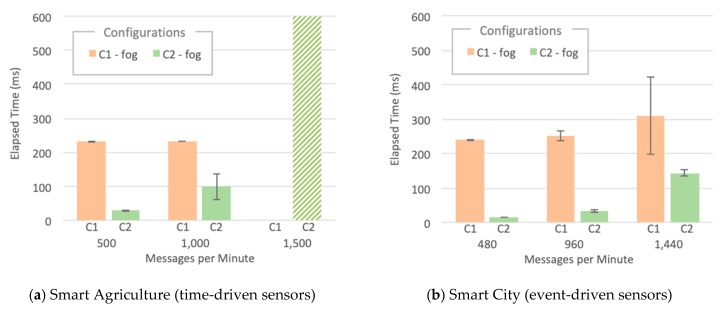
Device-to-Fog elapsed time for IoT smart applications.

In Figure 8, we see that for C1, most of the time budget is elapsed up to S3-Fog, whereas for C2, the time to reach and process data in the cloud dominates the path for both scenarios. This behavior comes from the ChirpStack processing time, present in C1 and absent in C2. For applications that require lower response times-like a Smart City scenario–the LoRaWAN 200 ms processing time might be unacceptable, which can make a case for a more straightforward LPWAN solution.

**Figure 9:**
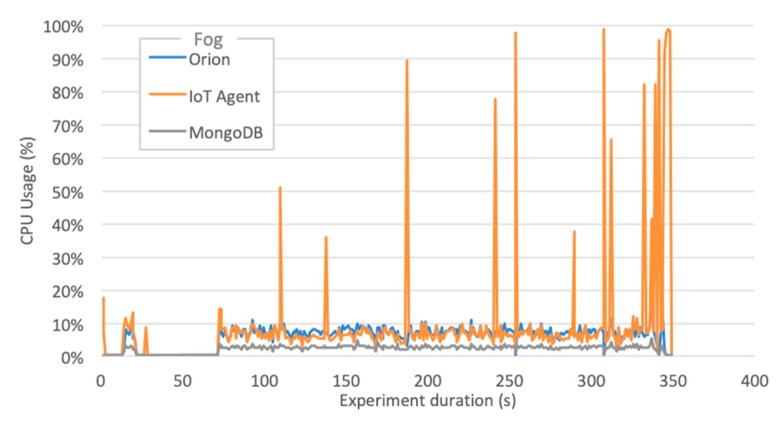
CPU usage of S3-Fog components-Configuration C1–Smart City.

**Figure 10:**
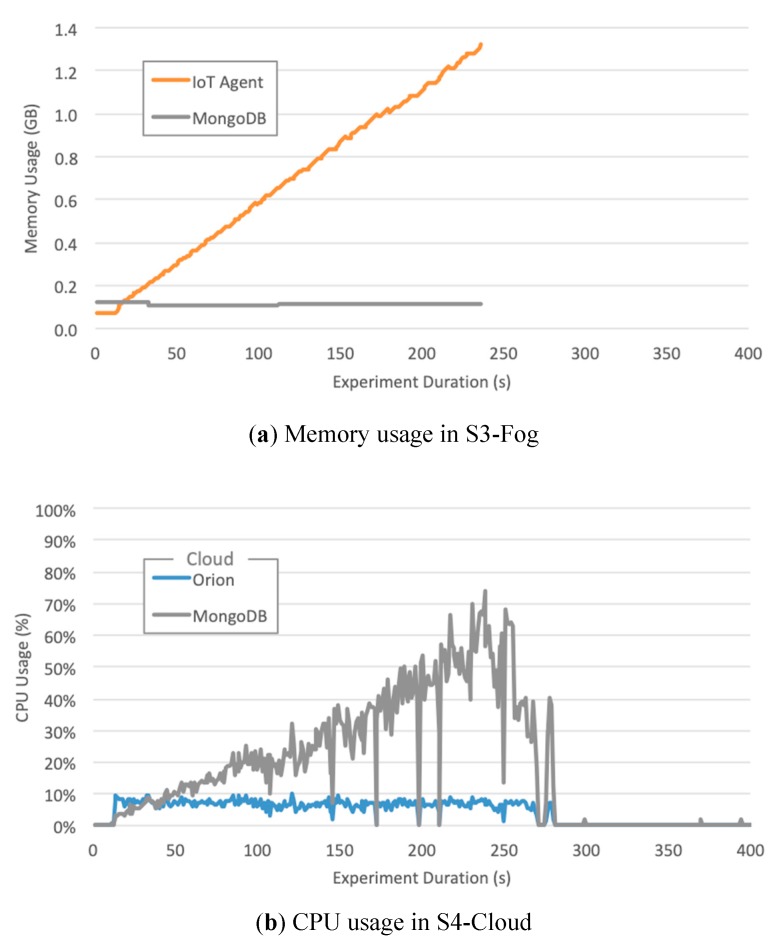
Cloud CPU Usage and Fog Memory Usage for Configuration C2 in a smart agriculture scenario in the experiment with 1500 messages/min.

We also measured CPU and RAM usage for all software components, located both in S3-Fog and S4-Cloud, which helped us to understand the behavior of the elapsed time metric. In all experiments that did not finish correctly, the cause was a failure in IoT Agent. However, it is relevant to notice that we used two different implementations: (a) a specially developed LoRaWAN IoT Agent; (b) the FIWARE official Ultralight 2.0 IoT Agent. Although both play the same role in converting IoT protocols into NGSI JSON and share the same programing language–JavaScript using Node.js–they differ in terms of performance and scalability. Some findings are worth highlighting:

- The LoRaWAN IoT Agent located at S3-Fog crashes in configuration C1 with the high workload for the smart city scenario due to spikes in CPU usage as depicted in Figure 9. A few seconds after a certain threshold of messages per second is reached, the CPU usage goes up to 100%, and this component presents a type of instability similar to a phase transition, leading it to crash soon after 341 s of the experiment.
- The Ultralight 2.0 IoT Agent located at both S3-Fog and S4-Cloud crashes in configurations C2, C3, and C5 with the high workload for the smart agriculture scenario due to a linear and constant increase in memory usage. Figure 10a shows it keeps allocation memory up to 239 s and crashes, which corresponds to the CPU usage of MongoDB, and also Orion, at S4-Cloud. Figure 10b shows that after the IoT Agent crashes, the CPU usage of MongoDB drops from more than 70% to almost zero after 40 s. In other words, the crash of the IoT Agent in S3-Fog is instead a consequence of another performance bottleneck located at S4-Cloud. The service time of the IoT Agent is shorter than the joint service time of Orion and MongoDB so that the former queues messages waiting for HTTP REST requests to be answered by the latter. In other words, the IoT Agent waits for a TCP ACK packet to free the memory allocated for the messages. As the message flow is constant in the smart agriculture scenario, the memory allocated by the IoT Agent increases boundlessly up to a point the operating system fails to provide more space, and it crashes. The high usage of CPU by MongoDB does not affect the performance of the platform since the IoT Agent crashes before the database starts to present significant problems.
- ChirpStack is a well-developed and stable software component that adequately uses system resources. The combined CPU Usage of all individual components that make up ChirpStack was around 40% for high workloads and 6% for low workloads, whereas the sum of memory usage was approximately 30 MB for high workloads and 22 MB for low workloads. Except for C1, all the configurations that crashed during the experiments were not based on LoRaWAN and consequently did not use ChirpStack. We conclude that, in addition to performing his LoRaWAN functions, the ChirpStack also works as a cushion for sensor data that softens message spikes that prevent IoT Agent to queue too many messages.
- The smart agriculture and smart city scenarios presented a similar performance for low and medium workloads. In those cases, the difference in the probability distribution for message generation did not impact the system performance, unlike for high workloads, where all experiments with Generic LPWAN (no ChirpStack) crashed for smart agriculture. On the other hand, in the smart city scenario, configurations C2 and C5 ran to the end of the experiment, and C3 was the only one with LoRaWAN that faced problems. This behavior indicates that the system can better handle traffic in bursts than in a constant flow of messages, because the time between bursts is enough for Orion to dequeue some messages and send ACKs back to the IoT Agent, freeing some memory in the process.

## 6. Discussion

### 6.1. Performance and Scalability Concerns

By analyzing the performance results shown in Section 5, we can understand that different deployment configurations of layered IoTecture components into staged IoTinuum places generate different hardware and software bottlenecks as far as the use of system resources is concerned. Also, that directly affects the critical application metric to determine the duration of data transfer (elapsed time) from its inception in S1-Thing by SenSE to its consumption in S4-Cloud by the consumer. Also, our results show that individual software components have different scalability behaviors that may change when they are connected to each other.

The complexity in understanding different tradeoffs complicates the choice of particular deployment configurations for specific IoT smart applications. In those cases, the help of an expert on the IoT platform will be required to correctly identify the most suitable set of components and configuration deployment for each scenario, considering typical workloads, as well as characteristics and constraints of the infrastructure and applications. We believe that our concepts related to architecting and deploying IoT smart applications, together with the practical performance analysis results, increased the understanding and awareness involved in the development and operation of a new breed of IoT-enabled systems. However, there is still the need for automating the process of application deployment, not only the static procedures for configuring and installing an application into the stages of IoTinuum, but also the dynamic analysis which may generate consequent system reconfigurations. The issue of static and dynamic configuration is currently an open research question.

Our experiments revealed that FIWARE general enablers—in their current version—are not designed for higher workload applications, which is a paradox because IoT Platforms should be intrinsically able to deal with thousands or millions of sensors simultaneously. When a FIWARE-based IoT platform is submitted to a workload of 1500 messages per minute, the system crashes due to the malfunctioning of the Ultralight IoT Agent. As IoT smart applications must handle a large number of connected devices sending messages continuously, 25 messages per second should not be considered a workload high enough to cause the system to fail. Therefore, even though Orion is considered a stable component, its joint use with the IoT Agent produces a bottleneck that puts a ceiling in the system scalability. This problem was not identified before in the literature because most FIWARE-based implementations are not deployed in large-scale scenarios–i.e., thousands of devices connected, as they focus on simple examples with just a few sensors, which does not stress the system enough to detect system bottlenecks. The scalability of IoT platforms—–FIWARE, in our case—to support the operation of applications using thousands or millions of sensors is another open research question.

When it comes to the six configurations analyzed in our experiments, we conclude that all of them have a practical use, depending on requirements, characteristics, constraints, and workload of smart applications and deployment scenarios. For example, in a scenario with stricter constraints on low latency, e.g., the smart city scenario for automated traffic lights, LoRaWAN powered by ChirpStack may not fulfill the demand. Also, such a configuration may require data to be consumed in the fog (S3-Fog), in order to avoid the latency of the network link between the edge device (S1-Thing) and the cloud (S4-Cloud). On the other hand, in smart agriculture, usually, there is no need for low latency. However, in some agriculture frontiers such as in the countryside of Brazil, highly unstable internet connections between S3-Fog and S4-Cloud are still common. Thus, application uptime can be increased, if it can survive to disconnections from S4-Cloud by processing simpler models in S3-Fog, e.g., configurations C1 and C2 where Orion and consumer are placed in S3-Fog. Increasing the understanding and awareness of design choices and best practices for deploying layered IoTecture components into staged IoTinuum is an open research question.

Configurations C3 and C4 rely on a lightweight S3-Fog only for communication purposes, since they have no way of consuming data locally. This choice lies in the fact farm infrastructure and connectivity with the Internet may be harnessed, but the local server installed in S3-Fog may continuously be under a low workload, so that its resources may be used in some creative ways, such as load balancing. This behavior was observed in the experiments with C4, which achieved better results compared to C6, where everything is processed in S4-Cloud. However, the opposite happened with C3, due to the poor scalability of IoT Agent. Therefore, using up idle capacity in S3-Fog requires careful analysis and an improved understanding of the behavior of specific software components. The tradeoffs in using fog resources are another open research question.

### 6.2. IoT Architectural Layers vs. Deployment Stages

IoTecture architectural layers paired together with IoTinuum deployment stages provide a simple and unambiguous way to bind software, hardware, and communication components to physical infrastructure in an IoT context. The physical location where a software component is executed has a high impact on the performance and brings different tradeoffs, as revealed by our performance analysis results. IoT smart applications have their specific characteristics and peculiarities regarding the location of physical infrastructure—stages S2-Mist, S3-Fog, and S4-Cloud—compared to traditional Web-based distributed applications.

There is no direct association between specific layers and stages, even though some choices make for sense than others. For example, components from layers L2-Transport, L3-Data, L4-Model, and even L5-Service can be deployed into an intermediary stage, such as configurations C1 and C2 that placed L2-Transport to L4-Model components into S3-Fog. On the other hand, all of them can be executed in S4-Cloud, such as configurations C5 and C6. Also, if S3-Fog implements a lightweight fog with no local L4-Model, there is no way to consume data locally from an edge device. Configurations based on lightweight S3-Fog for communication only are not able to consume data locally or decrease application latency, avoiding long internet delays. However, they can still be used for load balance purposes. Also, we evaluated configurations where ChirpStack is deployed in S3-Fog and S4-Cloud. Even though it could be installed in S2-Mist, we do not consider it a stable solution, since the mist node may not adequately fulfill the performance and reliability requirements of IoT smart applications.

Applications requiring strict compliance with low latency—such as smart city applications—should be designed having in mind layer L4-Service deployed in stages S2-Mist or 3S-Fog, as data can be consumed locally, thus, faster. On the other hand, for applications with an unstable connection between S1-Thing and S4-Cloud—e.g., smart agriculture applications—there is the need to store and process data in an intermediary stage, for increased uptime. Thus, such applications should have L3-Data components running in S2-Mist or S3-Fog. Here we presented some preliminary insights related to the deployment of layered components into staged locations, and further research is necessary to provide methods, mechanisms, algorithms, and best practices to avoid the need of a specialist and allow an automated deployment process.

### 6.3. Limitations and Future Work

This article does not exhaust the subject of designing IoT architectures and using them to guide and support the deployment of software, hardware, and networking components over distributed locations. Examples of limitations of this article that are left as future work include: (a) evaluating the performance of components located in higher layers, i.e., L4-Model and L5-Service; (b) automating the deployment process, and; (c) using different technology sets. The performance analysis of higher layer components has not inherent challenges, though the results would be highly dependent on specific applications. On the other hand, we have already started to develop an automated process for application deployment, both for generating static configurations at installation time, but also for dynamic on-the-fly adaptation.

The set of IoT platforms and LPWAN technologies used in the performance analysis study was fixed. Whereas FIWARE is a well-known IoT platform with a large user base, other ones might reveal different results and trade-offs, such as InterSCity [60], Konker [61], and ThingsBoard [62]. As for LPWAN technologies, our study only focused on LoRaWAN due to the do-it-yourself approach that facilitates installing and using it. On the other hand, experimenting with NB-IoT and SigFox with a large number of sensors will require the development of realistic emulation platforms based on extensive measurements with these LPWAN technologies to capture their behaviors. Besides, other open-source LoRaWAN servers, such as the one provided by The Things Networks, could be compared with ChirpStack.

## 7. Conclusions

Although IoT architectures play an essential role in the design of distributed smart applications, the development of IoT platforms derived from these architectures and their deployment into real scenarios yield different tradeoffs and thus require an improved understanding. We proposed a layered IoT architecture (called IoTecture) whose components are mapped to stages of an IoT computing continuum (called IoTinuum) in different deployment configurations. A performance analysis study with six configurations revealed that different deployment configurations of layered components into staged locations generate different hardware and software bottlenecks that affect system performance and scalability. Scalability problems that affect FIWARE components under high workloads were revealed.

## Figures and Tables

**Table 1:** Deployment Configurations.

Configuration	Fog Dilemma			Communication Technology
	Heavy-Weight	Light-Weight	No fog	
C1	✓			LoRaWAN
C2	✓			Generic LPWAN
C3		✓		Generic LPWAN
C4		✓		LoRaWAN
C5			✓	LoRaWAN
C6			✓	Generic LPWAN

**Table 2:** Software components for the performance analysis study.

Component	Layer	Implementation	Description
SenSE Sensor Simulator	L1-Device	SenSE Tool [56]	The Sensor Simulating Environment (SenSE) is an open-source large-scale IoT sensor data generator able to abstract real devices and to model different complex scenarios, such as smart cities [58] and smart agriculture [48]. SenSE is a traffic workload generator that emulates heterogeneous sensors representing tens of thousands of IoT devices, sending data simultaneously via MQTT. Although the sensors are synthetic, the traffic is real.
ChirpStack	L2-Transport	ChirpStack [34]	Implementation of LoRaWAN that can be installed in a private deployment. Composed by ChirpStack Network Server and ChirpStack Application Server, Redis and PostgreSQL databases, and Mosquitto MQTT Broker [59].
IoT Agent	L2-Transport	FIWARE	Translates specific data formats carried by IoT Protocols (such as Ultralight 2.0 over MQTT in this case) into standard FIWARE JSON NGSI model. IoT Agent stores its data in MongoDB. We considered two implementations of the IoT Agent: the FIWARE Ultralight 2.0 IoT Agent and a custom-made LoRaWAN IoT Agent. We developed the latter one since the existing one in the FIWARE repository is still unstable.
Orion	L3-Data	FIWARE	Orion is a publish/subscribe context broker with a central role in data distribution for the FIWARE platform. Orion works with entities defined in JSON NGSI and stores them directly in MongoDB.
Consumer	L4-Model	Specific purpose	A simple consumer of IoT data playing the role of a generic smart application model.

**Table 3:** Factors and levels for the performance analysis study.

Factor	Level
Scenario	Smart Agriculture–Smart City
Workload	
Smart Agriculture: soil sensor probes sending data every 10 min (time-driven sensors following a Constant distribution)	5000–10,000–15,000 (500–1000–1500 messages per minute)
Smart City: car arrival rate given in cars per second (event-driven sensors following a Poisson Distribution)	8–16–24 (480–960–1440 messages per minute)
Deployment configurations	C1–C2–C3–C4-C5-C6
